# How to accurately preoperative screen nipple-sparing mastectomy candidate—a nomogram for predicting nipple-areola complex involvement risk in breast cancer patients

**DOI:** 10.1186/s12957-023-02949-3

**Published:** 2023-03-01

**Authors:** Yuanbing Xu, Dai Pan, Yi Liu, Hanzhong Liu, Xing Sun, Wenjie Zhang, Chaohua Hu

**Affiliations:** 1 Department of Breast Surgery of Xiaogan Hospital affiliated to Wuhan University of Science and Technology, Xiaogan, 432100 Hubei Province China; 2 Department of Ultrasound of Xiaogan Hospital affiliated to Wuhan University of Science and Technology, Xiaogan, 432100 Hubei Province China; 3 Department of Cancer Statistics of Xiaogan Hospital affiliated to Wuhan University of Science and Technology, Xiaogan, 432100 Hubei Province China; 4 Department of Pathology of Xiaogan Hospital affiliated to Wuhan University of Science and Technology, Xiaogan, 432100 Hubei Province China

**Keywords:** Nipple-areola sparing mastectomy, Immediate breast reconstruction, Nipple-areola complex involvement, Predictive model, Nomogram

## Abstract

**Background:**

Nipple-sparing mastectomy (NSM) offers superior cosmetic outcomes and has been gaining wide acceptance. It has always been difficult to objectively quantify the risk of nipple-areola complex involvement (NACi). The goal was to develop a prediction model for clinical application.

**Methods:**

Patients who had a total mastectomy (TM) between January 2016 and January 2020 at a single institute formed the development cohort (*n* = 578) and those who had NSM + immediate breast reconstruction (IBR) between January 2020 and January 2021 formed the validation cohort (*n* = 112). The prediction model was developed using univariate and multivariate logistic regression studies. Based on NACi risk variables identified in the development cohort, a nomogram was created and evaluated in the validation cohort. Meanwhile, stratified analysis was performed based on the model’s risk levels and was combined with intraoperative frozen pathology (IFP) to optimize the model.

**Results:**

Tumor central location, clinical tumor size (CTS) > 4.0 cm, tumor-nipple distance (TND) ≤ 1.0 cm, clinical nodal status positive (cN +), and KI-67 ≥ 20% were revealed to be good predictive indicators for NACi. A nomogram based on these major clinicopathologic variables was employed to quantify preoperative NACi risk. The accuracy was verified internally and externally. The diagnostic accuracy of IFP was 92.9%, sensitivity was 64.3%, and specificity was 96.9% in the validation group. Stratified analysis was then performed based on model risk. The diagnostic accuracy rates of IFP and NACiPM in low-risk, intermediate-risk, and high-risk respectively were 96.0%, 93.3%, 83.9%, 61.3%, 66.7%, and 83.3%.

**Conclusion:**

We created a visual nomogram to predict NACi risk in breast cancer patients. The NACiPM can be used to distinguish the low, intermediate, and high risk of NAC before surgery. Combined with IFP, we can develop a decision-making system for the implementation of NSM.

## Background

The surgical treatment of breast cancer has evolved from maximal radical cure to maximal preservation of shape and function. Breast surgeons and patients pay attention to the expected survival time while taking into account the improvement of postoperative quality of life [1]. The surgical treatment of choice for most breast cancer patients is breast-conserving therapy. However, total mastectomy is still required in 30 to 40% of all breast cancer patients who undergo surgery [2, 3]. Nipple-sparing mastectomy (NSM), which evolved from skin-sparing mastectomy, is characterized by the preservation of the entire nipple-areola complex (NAC) and breast skin envelope despite the removal of the mammary tissue [4, 5]. NSM is a surgical procedure that improves patient satisfaction with cosmetic outcome [6].

However, previous studies have found that the rate of occult (pre)-malignant invasion of a clinically normal nipple in mastectomy specimens ranged from 0 to 58% [7, 8], implying that there was a risk of leaving an occult malignant tumor inside the NAC during NSM [9]. The main concern associated with NSM is the risk of local breast cancer recurrence at the retained NAC consequent to occult nipple involvement. As the oncology safety of NSM has been proven in multiple prospective and retrospective studies [1, 10–12], increasing numbers of patients with breast cancer are selecting NSM, the indications for NSM should be thoroughly reviewed.

NSM was provided to patients with a clinically normal NAC and no skin involvement. Although anomalous NAC clinical signs (inverted nipple, bloody nipple discharge, abnormal NAC skin, and so on) are frequently thought to be significant evidence of NAC invasion, there is presently no consistent and efficient preoperative evaluation technique for clinically occult NAC invasion. Previous research has found that tumor size, tumor-to-nipple distance, lymph node status, and tumor location are all clinical characteristics that may be linked to nipple-areola complex involvement (NACi) [9, 13]. Patient selection based on clinicopathologic criteria, on the other hand, is debatable, because a clinically negative NAC symptom can rule out occult nipple involvement with a high negative predictive value, even in individuals at high risk [14]. Furthermore, in clinical practice, differing measuring methods and categorization criteria have a significant impact on NACi outcomes. Tumor nipple distance (TND) is thought to be the most closely associated with NACi; however, different assessment methods, such as ultrasound (US), mammography (MG), and magnetic resonance imaging (MRI), have varied clinical implications. [15–17]. MRI is currently a more accurate testing approach for evaluating the abnormalities of NAC. Hirohito Seki and colleagues produced good results on the aforementioned topic and established two separate models for the NACi risk problem, the first of which was built based on clinical and imaging data and had an accuracy of 93.5%. The second model was based on MRI imaging data for prediction, with an accuracy of 96.0%, indicating that both models had a strong predictive ability [9, 18]. Patients who are unable to undertake or finish MRI exams within the effective period, on the other hand, are not in the minority. Based on this, we created a more easy, simple, and accurate visual model prediction tool using non-MRI data.

Nomograms based on clinical, imaging, and pathology data have been proven to provide more accurate predictions of tumor risk and treatment results for individual patients [19, 20]. We developed a nomogram based on preoperative indications to predict the likelihood of NACi in breast cancer patients in this study.

## Methods

### Patients

**Fig. 1:**
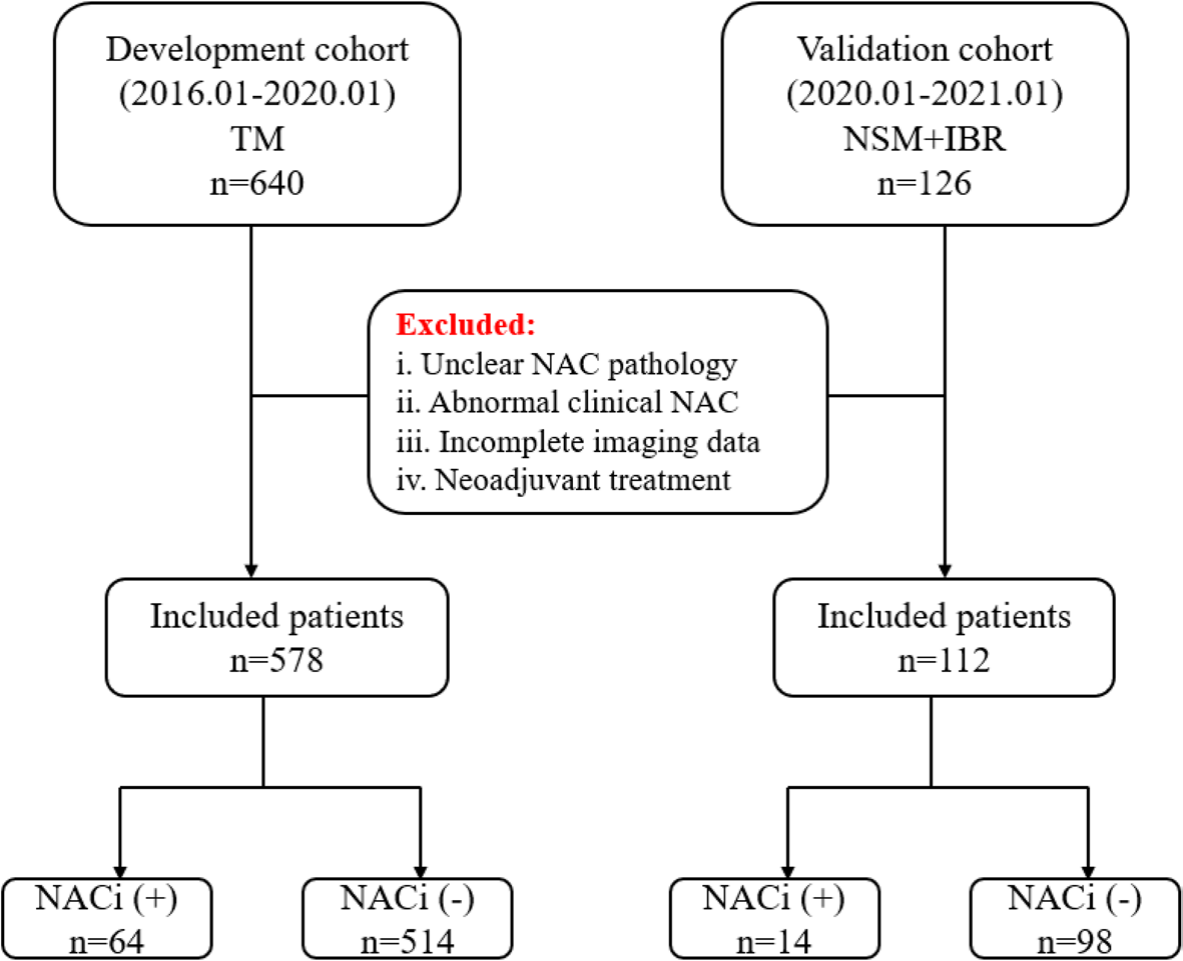
Study flowchart showing patients in development cohort and validation cohort

Data were collected from 766 patients with breast cancer treated with total mastectomy (TM)/NSM + immediate breast reconstruction (IBR) between January 2016 and January 2021 at a single institute. The exclusion criteria: (1) unclear NAC pathological results, (2) abnormal clinical NAC (inverted nipples, ulcer changes, eczematoid changes, and palpable masses behind the nipple) [21], (3) incomplete imaging data of MG and US, and (4) neoadjuvant chemotherapy. According to these criteria, a total of 578 patients who underwent TM between January 2016 and January 2020 formed the development cohort, and those who underwent NSM + IBR between January 2020 and January 2021 formed the validation cohort to confirm the model’s performance (Fig. 1).

Database research indicators include age, multifocal/single lesions, menopausal status, tumor location, nipple discharge, family history of cancer, clinical tumor size (CTS), TND, clinical nodal status (cN), mixed carcinoma in situ (MCIS), histological grade, pathological type, estrogen receptor (ER), progesterone receptor (PR), human-epidermal growth factor receptor 2 (HER2), and KI-67. Each variable can be accurately obtained preoperatively.

### Variable definitions and classification interpretation

To ensure study stability, we systematically identify and categorize included variables. When a cancer diagnosis has been made, multifocal tumors are characterized as two or more tumors in the same breast [22].

### Tumor location

The cylindrical breast tissue from under the areola to the chest wall under the NAC was defined as the central region [23]. Nipple discharge was defined as physiological nipple discharge and referred to as transparent discharge that was not bloody.

### CTS/TND

CTS was defined as the largest tumor diameter in unifocal instances and the maximum tumor diameter in multifocal situations. TND was defined as the lowest vertical distance from the tumor to the center of the ipsilateral nipple, with multifocal tumors having the shortest TND [24]. The maximum and minimum values of the two measuring techniques (MG/US) were recorded independently, with CTS recording the maximum and TND recording the minimum. A skilled radiologist analyzed every imaging data separately (Dr. Pan dai). Clinical physical examination and imaging results imply suspicious axillary lymph nodes, or preoperative ultrasound-guided needle aspiration cytology reveals axillary lymph node metastases, according to the positive definition of cN. Preoperative core needle biopsy (CNB) pathology results included information on MCIS, histological grade, pathological type, ER, PR, HER2, and KI-67. ER/PR positive was defined as the positive rate of tumor cells in immunohistochemical staining ≥ 1%, and the definition of HER-2 positive was HER2 3 + by immunohistochemistry or amplified by fluorescence in situ hybridization (FISH) or in situ hybridization (ISH). Ductal carcinoma in situ (DCIS) with or without infiltrating ductal carcinoma (IDC) was defined as MCIS.

### Surgical procedure

TM was approved in all patients in the development cohort, and NAC was given to a thorough pathological evaluation. NAC status was assessed in the validation cohort using intraoperative frozen pathology (IFP) and postoperative paraffin-fixed pathology. The subdermal glandular tissue was undermined in NAC, leaving 1 to 2 mm of the dermis intact. Then, after collecting a frozen section, a thin layer of glandular tissue was collected under the areola for evaluation [1]. If the nipple’s shape, color, and palpated characteristics were normal, and the NAC ducts were proven to be tumor-free in IFP, the NAC was suggested to be preserved. Skin or glandular cancer in situ (lobular carcinoma in situ was included), invasive carcinoma, vascular invasion, or Paget’s disease were all NAC-positive criteria. NAC negative criteria included intraductal papilloma, atypical hyperplasia, and mastopathy.

### Statistical analysis

The mean (± standard deviation, SD) of continuous variables is calculated and compared using an unpaired, two-tailed *t* test. Categorical variables were compared using the *χ*^2^ test or Fisher’s exact test. The clinical features data discrepancies between the development and validation cohorts were compared.

The development cohort was conducted to a univariate analysis to identify potential predictors of NAC positivity. Predefined variables were incorporated in a multivariate binary logistic regression analysis to determine corresponding regression coefficients with a 95% confidence interval (CI) for the development of the prediction model. These variables were used in a backward selection method to identify the independent risk factors for NAC involvement in a binary logistic regression analysis. Based on the backward stepwise regression method of R software, the effective variables were screened. A nomogram was developed to be a graphic representation of the model. For assessing the discriminative performance of the nomogram, the area under the curve (AUC) of the receiver operating characteristic (ROC) was measured. The calibration of the nomogram was performed internally in the development cohort and externally in the validation cohort, using a calibration plot with bootstrap sampling (*n* = 1000).

All statistical analyses were performed using SPSS 24.0 (IBM Corp, Armonk, NY, USA) or R version 4.1.2 software (R Core Team, 2021). All *p* values were two-sided, and *p* < 0.05 was considered significant.

## Results

### Patient characteristics

Data from 578 patients in the development cohort and 112 individuals in the validation cohort were included in this study. Table 1 summarizes the overall demographics of the patient population. Overall, the validation cohort after NSM + IBR had a NAC positive rate of 12.5% (14 out of 112) compared to 11.1% (64 out of 578) in the development cohort after TM. There was no significant difference in age, tumor location, nipple discharge, family history of tumor, CTS, TND, histological grade, pathological type, or molecular subtyping between the two groups (*p* > 0.05). The validation cohort, on the other hand, contained more postmenopausal, invasive cancer, and HER2-positive patients, and the difference was statistically significant (*p* < 0.05).

**Table 1:** Clinical characteristics of the patients in development and validation cohorts

Variables	Group, no. of patient		*P* value
	Development cohort (*n* = 578)	Validation cohort (*n* = 112)	
Age (years)			0.213
Mean ± SD	45.4 ± 6.2	42.2 ± 7.4	
Menopausal status			< 0.001*
*Premenopausal*	508 (87.9%)	82 (73.2%)	
*Postmenopausal*	70 (12.1%)	30 (26.8%)	
Multicentric/focal			0.297
*No*	466 (80.6%)	95 (84.8%)	
*Yes*	112 (19.4%)	17 (15.2%)	
Tumor location			0.147
*Central*	93 (16.1%)	12 (10.7%)	
*Peripheral*	485 (83.9%)	100(89.3%)	
Nipple discharge			0.134
*Yes*	82 (14.2%)	10 (8.9%)	
*No*	496 (85.8%)	102 (91.1%)	
Family history of cancer			0.509
*Yes*	84 (14.5%)	19 (17.0%)	
*No*	494 (85.5%)	93 (83.0%)	
CTS			0.317
≤ *4.0 cm*	496 (85.8%)	92 (82.1%)	
> *4.0 cm*	82 (14.2%)	20 (17.9%)	
TND			0.288
≤ 1.0 cm	67 (11.6%)	17 (15.2%)	
> 1.0 cm	511 (88.4%)	95 (84.8%)	
cN			0.688
*Positive*	118 (20.4%)	21 (18.8%)	
*Negative*	460 (79.6%)	91 (81.2%)	
MCIS			0.766
*Yes*	142 (24.6%)	29 (25.9%)	
*No*	436 (75.4%)	83 (74.1%)	
Histological grade			0.711
*G1*	115 (19.9%)	26 (23.2%)	
*G2/G3*	463 (80.1%)	86 (76.8%)	
Pathology type			0.004*
*Invasive*	312 (54.0%)	77 (68.8%)	
*Non-invasive*	266 (46.0%)	35 (31.2%)	
ER			0.140
*Positive*	415 (71.8%)	88 (78.6%)	
*Negative*	163 (28.2%)	24 (21.4%)	
PR			0.436
*Positive*	418 (72.3%)	85 (75.9%)	
*Negative*	160 (27.7%)	27 (24.1%)	
HER2			0.020*
*Positive*	154 (26.6%)	42 (28.6%)	
*Negative*	424 (73.4%)	70 (71.4%)	
KI-67			0.889
≥ *20%*	321 (55.5%)	63 (54.5%)	
< *20%*	257 (44.5%)	49 (45.5%)	
NACi			0.662
*Positive*	64 (11.1%)	14 (12.5%)	
*Negative*	514 (88.9%)	98 (87.5%)	

^*^Asterisks indicate statistically significant associations (*p* < 0.05)

### Univariate/multivariate analysis in the development

The development cohort’s potential predictors and the binary outcome, NAC involvement, were studied using univariable logistic regression analysis. Table 2 summarizes the results. NACi risk was associated with tumor location, CTS, TND, cN, and KI-67. In the development cohort, several factors, including multifocal, nipple discharge, histological grade, and pathological type had no statistically significant predictive value (*p* > 0.05). To forecast NACi risk, multivariable logistic regression was performed using the five factors presented in the nomogram.

**Table 2:** Univariate and multivariate logistic regression analysis in the development cohort

Variables	Univariate analysis		Multivariate analysis	
	OR (95%CI)	*P* value	OR (95%CI)	*P* value
Age, year	0.96 (0.92–1.00)	0.720		
Multifocal lesions				
* Yes*	1.91 (0.91–3.86)	0.076		
* No*	1.0			
Tumor location				
* Central*	2.09 (0.34–3.15)	0.001*	2.83 (1.48–5.36)	0.001*
* Peripheral*	1.0		1.0	
Menopausal status				
* Premenopausal*	1.74 (0.62–4.41)	0.261		
* Postmenopausal*	1.0			
Nipple discharge				
* Yes*	1.23 (0.55–3.03)	0.632		
* No*	1.0			
Family history of cancer				
* Yes*	1.50 (0.65–3.27)	0.322		
* No*	1.0			
CTS				
≤ *4.0 cm*	1.0 1.0			
> *4.0 cm*	4.44 (2.15–9.09)	< 0.001*	3.66 (1.85–7.15)	< 0.001*
TND				
≤ *1.0 cm*	6.55 (3.25–13.29)	< 0.001*	7.14 (3.48–14.28)	< 0.001*
> *1.0 cm*	1.0		1.0	
cN				
* Positive*	3.73 (1.93–7.21)	< 0.001*	3.58 (1.92–6.67)	< 0.001*
* Negative*	1.0		1.0	
MCIS				
* Yes*	1.21 (0.60–2.38)	0.589		
* No*	1.0			
Histological grade				
* G1*	1.0			
* G2/G3*	1.73 (0.95–3.17)	0.072		
Pathology type				
* Invasive*	1.0			
* Non-invasive*	1.26 (0.67–2.38)	0.475		
ER				
* Positive*	1.0			
* Negative*	0.82 (0.28–2.45)	0.714		
PR				
* Positive*	1.0			
* Negative*	0.60 (0.21–1.77)	0.350		
HER2				
* Positive*	1.58 (0.78–3.13)	0.197		
* Negative*	1.0			
KI-67				
< *20%*	1.0		1.0	
≥ *20%*	2.47 (1.35–4.63)	0.003*	2.00 (1.12–3.63)	0.019*

^*^Asterisks indicate statistically significant associations (*p* < 0.05)

### Nomogram of development and validation

Based on the backward stepwise regression method of R software, the effective variables were screened. TND was the most effective factor for predicting NACi risk, (OR 7.14, 95% CI 3.48–14.28; *p* < 0.001). Central location tumor (OR 2.83, 95% CI 1.48–5.36; *p* < 0.001) and TND were highly correlated. According to the results, larger tumor size patients (> 4.0 cm) were more inclined to add the risk of NACi than small tumor size (OR 3.66, 95% CI 1.85–7.15; *p* < 0.001). Patients with cN + breast cancer were more likely to increase the risk of NACi than patients with cN- (OR 3.58, 95% CI 1.92–6.67;* p* < 0.001). In contrast, patients with the low KI-67 disease were more likely to reduce the risk of NACi than patients with high KI-67 (OR 2.00, 95% CI 1.12–3.63; *p* = 0.019).

**Fig. 2:**
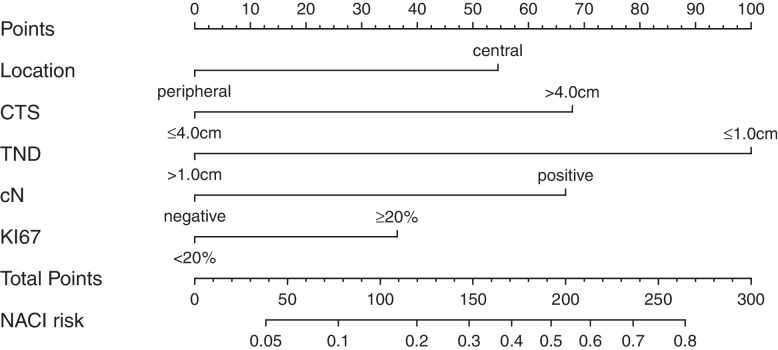
A nomogram to predict the risk of NAC involvement

A nomogram based on the independent predictors identified in the multivariate logistic regression analysis, including tumor location, CTS, TND, cN, and KI-67 to predict the risk of NACi was drawn (Fig. 2). The optimal cutoff point calculated by the model based on the development cohort was 0.084, with a sensitivity of 67.5% and a specificity of 76.6.%. If the nomogram score was < 80 points, the NACi risk was less than 10%, and the 190 points NACi risk was around 50%.

**Fig. 3:**
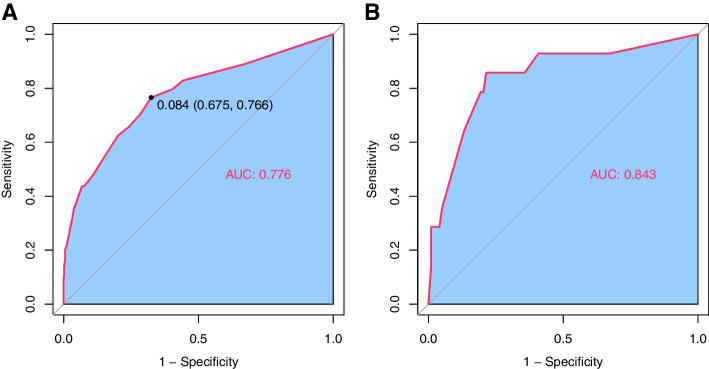
Area under the ROC curves of the nomogram to predict the risk of NACi in the **A** development cohort and **B** validation cohort. The optimal cutoff point calculated by the model based on the development cohort was 0.084, with a sensitivity of 67.5% and a specificity of 76.6.%

**Fig. 4:**
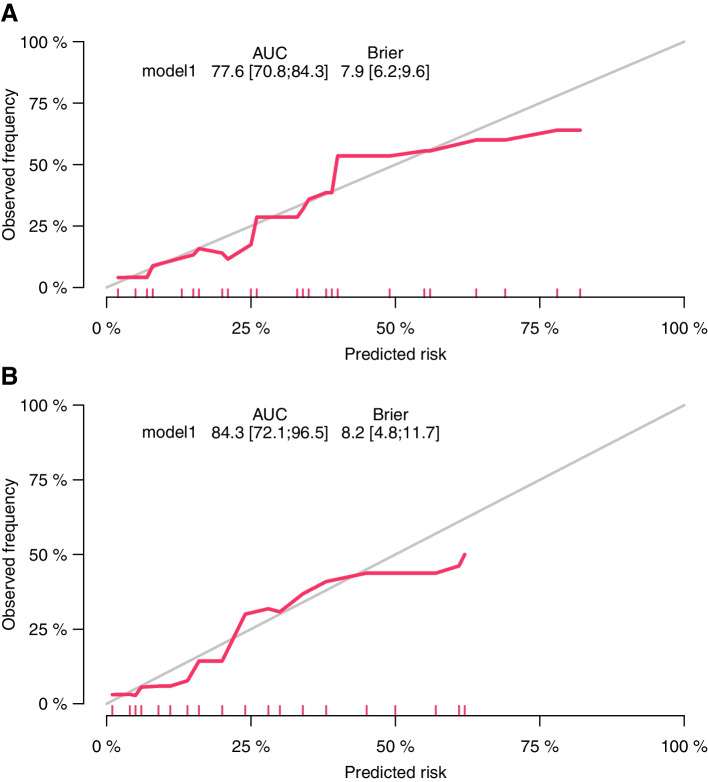
Calibration of the nomogram to predict the risk of NACi in the **A** development cohort and **B** validation cohorts

ROC analysis was performed to validate the nomogram internally in the development cohort (Fig. 3A) and externally in the validation cohort (Fig. 3B), with AUC values of 0.776 (95% CI 0.708–0.843) and 0.843 (95% CI 0.721–0.965) respectively, suggesting that it had a good predictive ability. The increase of AUC from 0.776 to 0.843 (TM to NSM), which due to there being substantial of cases subnipple biopsy-proven NACi, but final pathology showed no direct nipple invasion. A calibration curve was generated for the evaluation of calibration (Fig. 4A, B). There was a satisfactory agreement between the predicted probability and the observed probability, according to an administered Hosmer–Lemeshow test (Hosmer–Lemeshow test in the development cohort: chi-square = 4.338, *p* = 0.502; Hosmer–Lemeshow test in the validation cohort: chi-square = 2.683, *p* = 0.749).

### Prospective applications of the nomogram

#### Case analysis

**Fig. 5:**
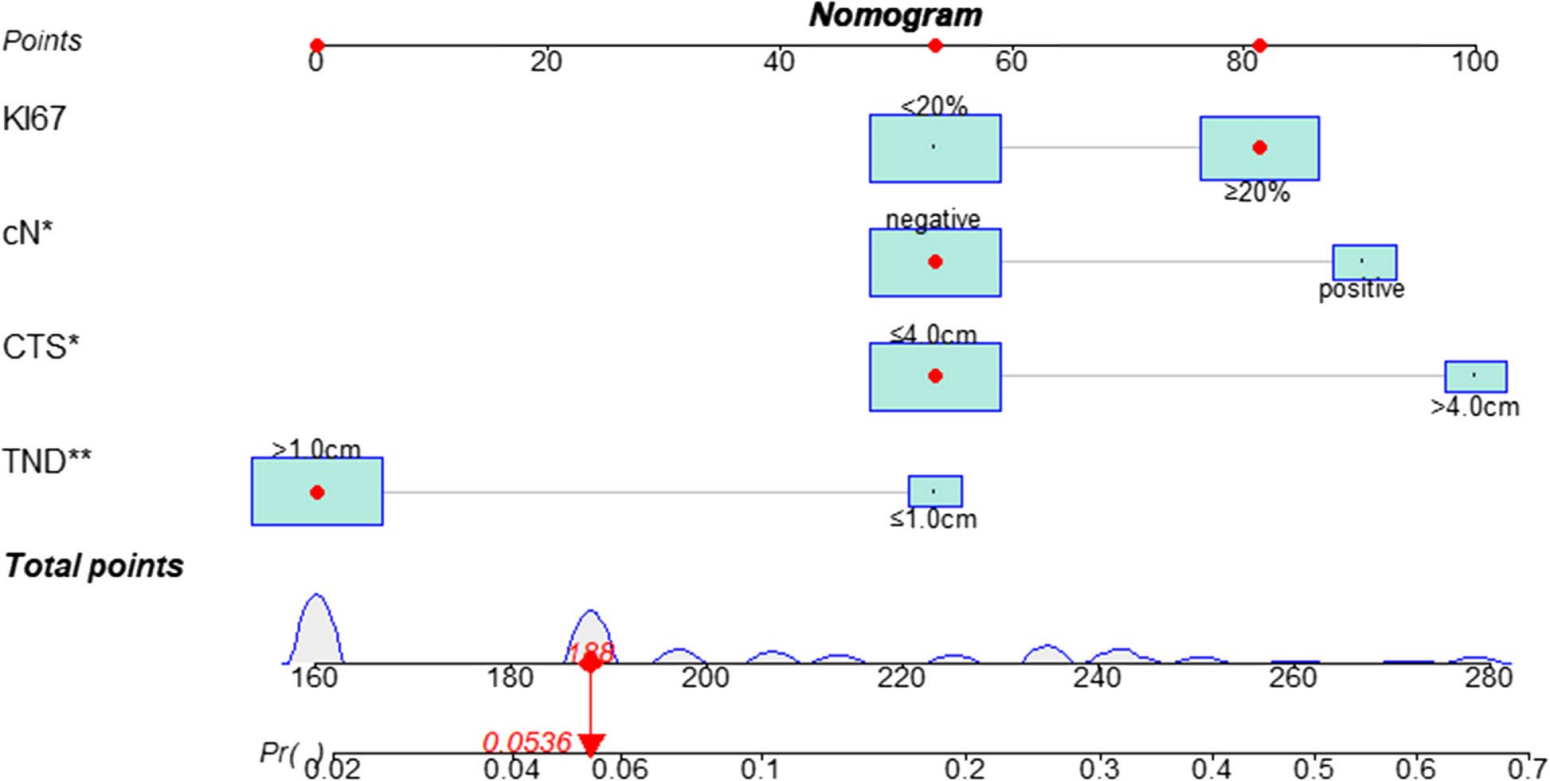
Case-specific analysis: ki67, 35%; cN( −); CTS: 3.2 cm; TND: 2.4 cm. In the nomogram, a total of 188, NACi risk = 0.0536. The actual NAC pathology was negative

A 45-year-old patient was diagnosed with left-sided breast cancer. Imagine examination before surgery showed: CTS was 4.2*2.5 cm (MG), 3.50*1.05 cm (US), tumor central location, and TND was 1.21 cm (MG), 1.54 cm (US), cN ( −), Preoperative CNB biopsy: invasive ductal carcinoma, G2, ER +  + (85%), PR (+ , 50%), HER-2 ( −), and KI67 (35%). NACi risk can be predicted by nomogram was 5.36%, and postoperative pathology confirmed NAC negative (Fig. 5).

#### Stratified analysis

IFP of NAC was performed for the cases in the validation group. The results were shown in Table 3, and the diagnostic accuracy of IFP was 92.9%, sensitivity was 64.3%, specificity was 96.9%, the false-positive rate was 3.1%, and the false-negative rate was 35.7%.

**Table 3:** Correlation between IFP and NAC involvement in permanent pathological specimens

		Actual NAC involvement		Total
		NACi( +)	NACi( −)	
IFP	NACi( +)	9	3	12
	NACi( −)	5	95	100
Total		14	98	112

Sensitivity 64.3%, specificity 96.9%, FPR 3.1%, FNR 35.7%, accuracy 92.9%

*Abbreviations*: *FNR*, false-negative rate; *FPR*, false-positive rate; *NACi*, nipple-areola complex involvement; *IFP*, intraoperative frozen pathology

**Fig. 6:**
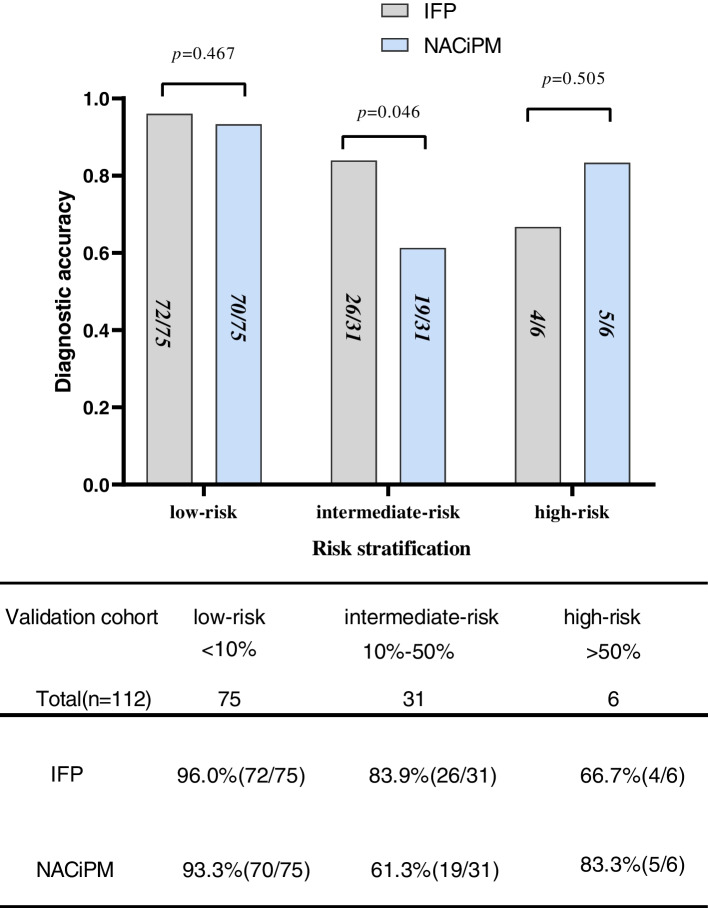
Stratified analysis. The diagnostic accuracy rates of IFP and NACiPM in low risk, intermediate risk, and high risk respectively were 96.0% (72/75) and 93.3% (70/75), 83.9% (26/31) and 61.3% (19/31), and 66.7% (4/6) and 83.3% (5/6)

The criterion for NACi prediction model (NACiPM) positivity was defined as the NACi risk corresponding to the best cutoff value of the nomogram. We further combined the NACiPM with IFP and stratified analysis according to low-risk (NACi risk < 10%); intermediate-risk (10–50%); high risk (> 50%). The diagnostic accuracy rates of IFP and NACiPM in low risk, intermediate risk, and high risk respectively were 96.0% (72/75), 93.3% (70/75), 83.9% (26/31), 61.3% (19/31), 66.7% (4/6), and 83.3% (5/6). The diagnostic accuracy of IFP and NACiPM in the low-risk group and the high-risk group was comparable (*p* = 0.505, *p* = 0.467), and the accuracy of IFP in the intermediate-risk group was higher, the difference was statistically significant (*p* = 0.046) (Fig. 6).

#### Optimize process

**Fig. 7:**
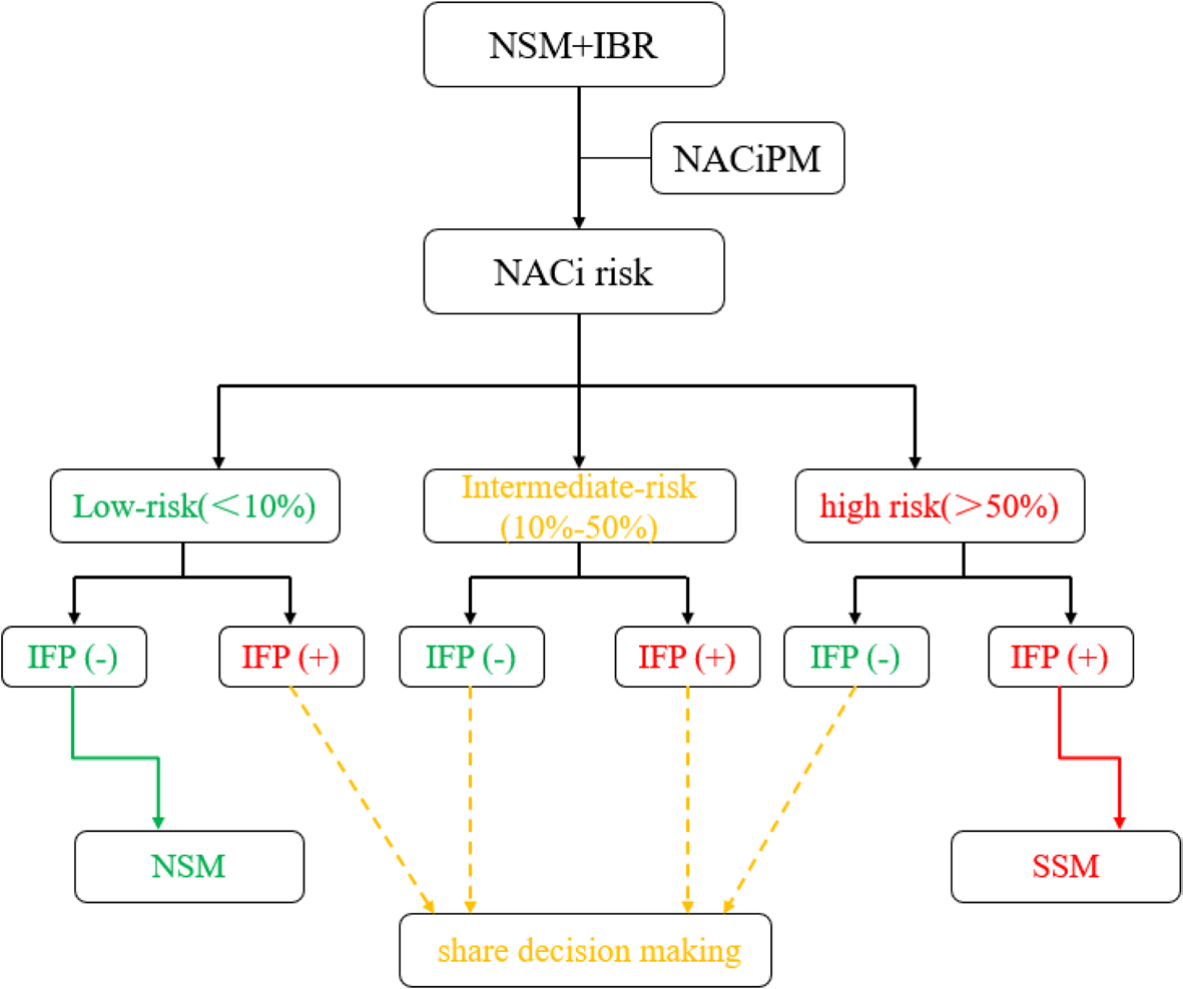
For patients with NACi risk < 10% and IFP ( −), NSM would be recommended. While patients with NACi risk > 50% and IFP ( +), SSM would be suggested. Patient-physician shared decision making would be recommended in the other situation

In combination with the NACiPM and IFP results, we propose that the following procedures be optimized for patients undergoing NSM + IBR. For patients with NACi risk < 10% and IFP ( −), NSM would be recommended. While patients with NACi risk > 50% and IFP ( +), SSM would be suggested. Patient-physician shared decision-making would be recommended in the rest of the situation (Fig. 7).

## Discussion

At present, the commonly used clinical prediction tools for NACi include clinical physical examination, imaging, and pathological evaluation. Although the criteria used to select NSM for IBR have broadened over time, the consensus has not yet been reached regarding this issue [1, 9, 15, 17, 18]. Previous studies indicated that tumor size, TND, lymph node status, lymphovascular invasion, and tumor location were closely related to NAC invasion. Pathological indicators such as pathological type, histological grade, ER/PR, HER2, and KI-67 showed great differences in different studies [25–27]. However, predicting NACi risk using these recommendations alone is problematic since some of the factors cannot be evaluated preoperatively.

There is an urgent need for really simple and reliable prediction models that can be used in clinical practice. Hirohito Seki and colleagues have carried out a series of studies and achieved good prediction results [9, 18]. CTS, TND by MG/MRI, nipple contrast findings by MRI, tumor in a central portion, multicentric/focal lesion, and cN positive were significantly associated with the presence of NAC involvement. Part of the experimental results was validated in our study. And they developed a more accurate preoperative prediction model based on enhanced breast MRI. The experimental results showed that the model achieved sensitivity 89.4%, specificity 97.6%, PPV 89.4%, NPV 97.6%, FNR 10.6%, FPR 2.4%, and accuracy 96.0%. Unfortunately, it did not achieve the desired results owing to radiologists having great differences in the assessment of some indicators. Nevertheless, most clinicians believe that MRI may not be so necessary for patients who intend to perform NSM. In addition, the high expenses and time costs of MRI limit the popularity of the model [28]. Therefore, we have developed a prediction model based on general clinical imaging and pathological indicators. This nomogram prediction model makes the results more concise and visual, which has strong clinical operability.

Published studies show larger CTS and smaller TND are always more likely to lead to NAC positive, but different tumors in imaging, especially when combined with carcinoma in situ components, show great differences [26, 29]. However, there was no consistent conclusion in our study, which may be due to the underestimation of preoperative hollow needle aspiration in the diagnosis of CIS. To achieve better consistency, we improve the CTS/TND measurement idea in this study. MG and US measurements were recorded separately, in which CTS recorded the maximum value and TND recorded the minimum value of the two measurement methods. In the literature, the optimal cutoff distance varies. The optimal CTS cutoff value achieved with the ROC curve analysis in this study was 3.78 cm, which was consistent with earlier research [9, 30]. In this investigation, we used a threshold value of 4.0 cm for grouping. At the same time, while the optimal TND cutoff value was 1.2 cm, we used 1.0 cm for grouping, which was consistent with the majority of the literature [18, 31].

In this study, according to the binary logistic regression model, it was concluded that CTS > 4.0 cm, TND ≤ 1.0 cm, cN( +), tumor central location, and ki-67 ≥ 20% were independent predictors of NACi risk. Although we analyzed more histopathological variables, we did not find significant associations between these factors and NAC involvement. The nomogram was produced drawn on different weight coefficients, and the model accuracy rate was 0.776 in the development group and 0.843 in the validation group. This may be the first visualization model for NACi risk prediction to our knowledge, the internal validation results of both groups were good. This model can visually analyze the data well, score each independent predictor separately, and calculate predicted probability. This simple and easy visualization process greatly facilitates preoperative doctor-patient dialogue and the prediction of risk assessment.

The occult positive rate of NACi was 11.1% (64/578) in the development group and 12.5% (14/112) in the validation group, with no significant difference between the two groups. This indirectly demonstrates that IFP can accurately estimate the potential of NACi [32, 33]. The diagnostic accuracy of IFP was 92.9%, sensitivity was 64.3%, specificity was 96.9%, the false-positive rate was 3.1%, and the false-negative rate was 35.7%. We noticed that the false-negative rate was rather high, with the false-negative rate of IFP reported in prior literature ranging from 0.7 to 54.5% from 2008 to 2021 [18, 34–36]. As a result, other surgeons did not approve that IFP was required [18, 37].

We further conducted stratified analysis according to the model risk and set NACi risk < 10% as low-risk, 10–50% as intermediate-risk, and > 50% as high-risk. According to our research data, in the low-risk group, both IFP and the model had a certain false-negative rate, and IFP was higher, and the false-positive rate of both was very low. This may explain that the model can be complementary to IFP improvements in some patients. Because a higher false-positive rate means that more NAC may be resected incorrectly, the particularly pernicious false-positive rate is significantly greater than that of reoperation due to false negatives.

In our study, the results suggested that the IFP diagnosis was negative in 5 patients, while the postoperative pathology was positive. One case of DCIS and 4 cases of lobular carcinoma in situ (LCIS) was diagnosed by postoperative pathology. For NAC diagnosed as LCIS, clinical management is relatively difficult, if LCIS is regarded as a precursor of invasive carcinoma [38]; thus, the NAC should be excised. Rachel E.K [32] approved that irrespective of whether LCIS is viewed as a “marker” or as a “precursor” lesion, recommend NAC resection in such cases since there is inadequate follow-up data recognizing that others may hold a contrary view. If LCIS is excluded, then the FNP of IFP is 7.14% in our study (versus 35.7% if LCIS is included).

The model’s value was that it could appropriately screen the absolute low-risk and high-risk groups of NAC involvement preoperatively. For patients with NACi risk < 10% and IFP ( −) can implement NSM securely, while patients with NACi risk > 50% and IFP ( +) should be recommended SSM. For patients with intermediate-risk, or NACi risk < 10% and IFP ( +), or NACi risk > 50% and IFP ( −), these three scenarios should recommend patient-physician shared decision making. This prediction model is simple to calculate, which greatly facilitates the judgment of clinical surgeons in the assessment of the positive risk of NACi.

## Conclusions

In this study, we developed a NACiPM based on non-MRI data, an accurate and visual predictive model in NSM + IBR. The NACiPM can be used to distinguish the low, intermediate, and high risk of NAC before surgery. Combined with IFP, we can develop a decision-making system for the implementation of NSM.

## Data Availability

The datasets used during the current study are available from the corresponding.

## Abbreviations

NAC: Nipple-areola complex
IBR: Immediate breast reconstruction
TM: Total mastectomy
NACi: Nipple-areola complex involvement
NSM: Nipple-sparing mastectomy
TND: Tumor-nipple distance
CTS: Clinical tumor size
cN: Clinical nodal status
MG: Mammography
MRI: Magnetic resonance imaging
MCIS: Mixed carcinoma in situ
ER: Estrogen receptor
PR: Progesterone receptor
Her2: Human-epidermal growth factor receptor 2
DCIS: Ductal carcinoma in situ
IDC: Infiltrating ductal carcinoma
LCIS: Lobular carcinoma in situ
CNB: Core needle biopsy
OR: Odds ratio
CI: Confidence interval
IFP: Intraoperative frozen pathology
NACiPM: NACi prediction model
